# Genome Estimation and Phytochemical Compound Identification in the Leaves and Callus of *Abrus precatorius*: A Locally Endangered Plant from the Flora of Saudi Arabia

**DOI:** 10.3390/plants11040567

**Published:** 2022-02-21

**Authors:** Fahad Al-Qurainy, Mohamed Tarroum, Salim Khan, Mohammad Nadeem, Abdel-Rhman Z. Gaafar, Saleh Alansi, Norah S. Alfarraj

**Affiliations:** Department of Botany and Microbiology, College of Science bldg5, King Saud University, Riyadh 11451, Saudi Arabia; falqurainy@ksu.edu.sa (F.A.-Q.); skhan2@ksu.edu.sa (S.K.); mnadeem@ksu.edu.sa (M.N.); agaafar@ksu.edu.sa (A.-R.Z.G.); salansi@ksu.edu.sa (S.A.); 438203416@student.ksu.edu.sa (N.S.A.)

**Keywords:** *Abrus precatorius*, callus induction, flow cytometry, phytochemical compounds

## Abstract

*Abrus precatorius* is considered to be a valuable source of natural products for the development of drugs against various diseases. Herein, the genome size and phytochemical compounds in the leaves and callus of *A. precatorius* were evaluated. The endangered *A. precatorius* was collected from the Al-Baha mountains, Saudi Arabia and identified based on the phylogenetic analysis of a DNA sequence amplified by ITS1 and ITS4 primers. The callus was induced by the culture of stem explants onto Murashige and Skoog medium (MS) supplemented with various combinations of 2,4-dichlorophenoxyacetic acid (2,4D) and 6-Benzylaminopurine (BAP). The callus with the highest fresh weight (2.03 g) was obtained in the medium containing 0.5µM BA and 5 µM 2,4-D after 8 weeks of culture; thus, the callus of this combination was selected for the genome estimation and phytochemical compound extraction. The genetic stability of the leaves from the donor as well as in the regenerated callus was analyzed by flow cytometry with optimized tomato (2C = 1.96 pg) as an external reference standard. The 2C DNA content was estimated to 1.810 pg ± 0.008 and 1.813 pg ± 0.004 for the leaves and callus, respectively. Then, the total phenol and total flavonoid contents in the methanol extract of the callus and leaves were measured using a spectrophotometer and the High-performance liquid chromatography (HPLC ) methods. The results showed that the methanolic extract of the leaves was higher in total phenols and total flavonoids than the callus extract. Finally, the extracts of callus and leaves were analyzed for phytochemical compound through the Gas chromatography and Mass spectroscopy (GC-MS). A total of 22 and 28 compounds were detected in the callus and leaves, respectively. The comparative analysis showed that 12 compounds of the secondary metabolites were present in both extracts.

## 1. Introduction

Saudi Arabia is one of the richest biodiversity areas on the Arabian Peninsula that comprises a very important genetic resource. A total of 2250 species belonging to 142 families are represented in the flora of the Kingdom of Saudi Arabia. Of these, there are 242 endemic and 600 rare and endangered species [1]. Rare and endangered species are classified according to the percentage of frequency. For instance, a species is considered as endangered when one or more of its populations have declined, whereas a species becoming extinct is considered as rare [2]. *Abrus Precatorius* is one of the endangered plant species of the flora of Saudi Arabia [3,4]. In the Al Baha region of Saudi Arabia, Al-Khulaidi et al. [2] noted that the frequency percentage and the density per hectare of *Abrus Precatorius* were 0.31 and 0.05, respectively. Moreover, in Jabal Fayfa, southwest of Saudi Arabia, *Abrus Precatorius* is classified as a non-endemic-endangered species [5]. *Abrus Precatorius* is a herbaceous plant of the Fabaceae family. Its leaves are compound pinnate turgid, oblong, obtuse, and truncated at both ends with seven to twenty-four pairs of leaflets. The flowers are found in auxiliary racemes with a pink or pinkish-white color; the grains are found in pods that are 1.5 to 5.0 cm long, turgid, oblong, and appressed hairy, with a pointed and deflected beak, silky texture, and red poisonous seeds with a black mark at the base [6]. The plant is used in some traditional medicine, and it is noted to have a wide range of therapeutic effects, such as antibacterial, antifungal, antitumor, analgesic, antispasmodic, antidiabetic, antiserotonergic, antimigraine, and anti-inflammatory effects, including in the treatment of ulcers, wounds, scratches of the throat, and sores. It is also considered to be a valuable source of natural products for the development of drugs against various diseases and for the development of industrial products [7]. In Nigeria, the leaves of *A. precatorius* are used for the treatment of several diseases, including malaria, typhoid, respiratory tract infections, and hepatitis. The leaves are taken orally as a medicine, since they do not contain as much of the deadly abrin component as is found in the seeds. In addition, it was reported that the fresh leaves can be pressed onto the gum tissue to treat mouth pain and skin cancer [8]. Phytochemical studies of the aqueous leaf extract of *A. precatorius* have shown that the compounds identified in the extract comprise a complex mixture of several classes of phenolic compounds, terpenoids, and steroids [9]. According to [6], the compounds found in *A. precatorius* leaf extract were identified as abrine, trigonelline, abruslactone A, hemiphloin, abrusoside A, abrusoside B, abrusoside C, abrusoside D, arabinose, galactose, xylose, choline, hypaphorine, precatorine glycyrrhizin, montanyl alcohol, inositol, D monomethyl ether, and pinitol. The authors of [10] recommended *A. precatorius* as a plant of phytopharmaceutical importance on account of its abundant major phyto-compounds that can be utilized by drug designers following their appropriate isolation and characterization.

The propagation of *A. precatorius* by seeds is difficult because of their hard seed coat, a characteristic which explains its dispersed distribution. Therefore, it is important to develop this medicinally important taxon through in vitro propagation to save it from a further decrease, and to meet the demand of the traditional medicine industry [11]. In addition, in vitro propagation could be useful as a potential technology to increase the production of medicinal plants and to preserve rare and endangered species. Although there are minimal chances of instability in the regenerated plants, which are directly formed from cultured tissue, there are probabilities of cell mutations and the genetic variability of somatic embryo-regenerated plants. These genomic variations can be triggered by different factors, such as the age of the culture, continuous exposure to plant growth regulators

(PGRs) such as 2,4-dichlorophenoxyacetic acid (2,4-D), 6-Benzylaminopurine (BAP), and α-naphthaleneacetic acid (NAA), and adaptation to the stress of in vitro conditions [12]. Thus, the in vitro cultures must be personalized to avoid changes in the plant genome. The detection of structural chromosomal aberrations as well as changes in chromosome number, including polyploidy and aneuploidy, allow direct information regarding critical genomic changes and the analysis of the level of variation in the regenerated plants. Flow cytometry is a rapid and efficient method for nuclear DNA content estimation in thousands of cells and has been widely used for the rapid screening of ploidy levels in regenerated plants [13,14]. Additionally, flow cytometry was used for several different purposes, including the determination of the amount of species-specific DNA, the analysis of cell cycle activity in different tissues, and the measurement of endopolyploidization levels [15,16,17].

This study is perhaps the first 2C DNA estimation for the medicinal plant *A. precatorius*. It also investigated the phytochemical compounds in the extracts of the leaves and callus through different methods.

## 2. Results

### 2.1. Molecular Phylogenetic Identification of Abrus precatorius

**Figure 1:**
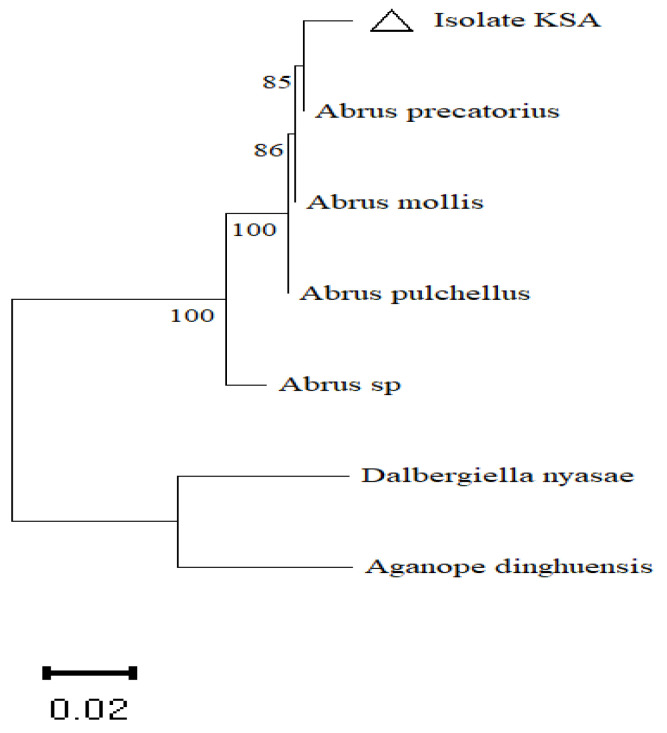
Identification of *Abrus precatorius* through the amplification of the internal transcribed spacer (ITS) region. Phylogenetic tree was constructed using MEGA X software and neighbor-joining methods by bootstrapping 1000 times. Scale bar: 0.02 substitutions per nucleotide.

To identify the species of our collected plant, the internal transcribed spacer (ITS) region was amplified using ITS1 and ITS4 primers. After assembling both the reverse and forward sequences, screening in the NCBI GenBank by the use of the BLASTN tool revealed the highest identity of 97.66% with *A. precatorius* (accession number JN407458.1), which was previously published. Moreover, the neighbor-joining method based on the Kimura two parameter model used to estimate a matrix of pairwise distances indicated that the isolate plant was likely *A. precatorius*. To root the in-group of the *Abrus* species in the constructed tree, the two plants species *Dalbergiella nyasae* and *Aganope dinghuensis* were selected as an outgroup (Figure 1).

### 2.2. Callus Induction

**Figure 2:**
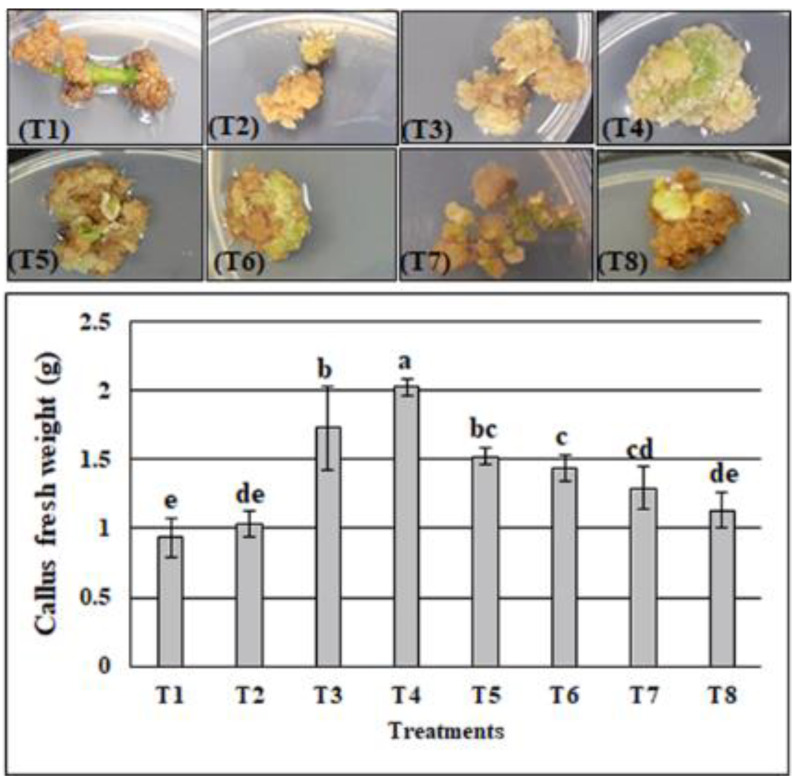
Effect of plant growth regulators on callus morphology and fresh weight after 8 weeks of cultures (T1—0.5 µM BA + 0.5 µM 2,4-D; T2—0.5 µM BA + 1 µM 2,4-D; T3—0.5 µM BA + 2.5 µM 2,4-D; T4—0.5 µM BA + 5 µM 2,4-D; T5—1 µM BA + 0.5 µM 2,4-D; T6—2.5 µM BA + 0.5 µM 2,4-D; T7—5 µM BA + 0.5 µM 2,4-D; T8—5 µM BA + 5 µM 2,4-D; BA—6-benzyladenine; 2,4-D—2,4-dichlorophenoxyacetic acid). Values are the mean of three replicates ± standard deviation (S.D.) different letters on bars indicate the significant differences according to Duncan’s test (*p* < 0.05).

For the callus induction, the stem explants of *A. precatorius* were cultured in Murashige and Skoog MS medium amended with BA and 2,4-D at different concentrations (0.5, 1.0, 2.5, and 5 µM). All used combinations were able to induce the callus at the cut surfaces of the explants. However, callus morphology and callus fresh weight varied depending on phytohormone combinations. After 8 weeks of culture, the callus with the highest fresh weight (2.03 g) was observed in the medium containing 0.5 µM BA and 5 µM 2,4-D; this was significantly different from the other combinations, which showed 0.93, 1.035, 173, 1.52, 1.43, 1.29, and 1.13 g for T1, T2, T3, T5, T6, T7, and T8, respectively (Figure 2). On the other hand, the callus derived from the T3 and T4 combinations was white and friable, while that derived from the other treatments had a brown color.

Based on these findings, only the callus induced from the T4 combination was used for the genome estimation and for the extraction of phytochemical compounds.

### 2.3. DNA Content Estimation through Flow Cytometric Analysis

**Figure 3:**
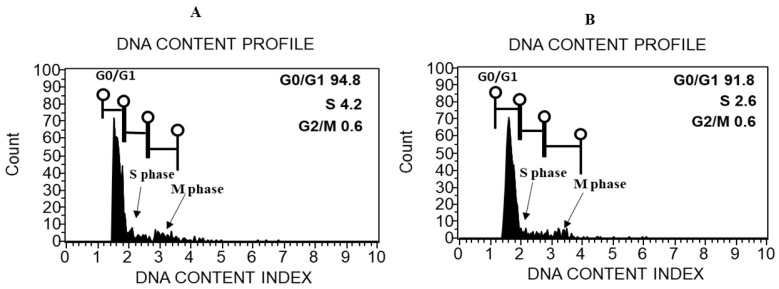
Flow cytometric histograms of the relative DNA content obtained from nuclei of *Abrus precatorius*: (**A**) nuclei extracted from the donor plant and (**B**) nuclei extracted from the callus. G0, G1, S, G2 and M indicate the interphase and the mitotic phase of the cell cycle.

The measurement of the cell cycle compartments G0/G1 (2C), S, and G2/M (4C) was performed by the use of a flow cytometer to estimate the DNA content index and the ploidy level of the induced callus and leaves from the donor *A. precatorious* plant. The DNA content index histograms of both the callus and the leaves had similar fluorescence intensities corresponding to 2×. The mean peaks of fluorescence intensity generated from the callus and leaves (G0/G1) were 91.8 and 94.8, respectively. Using tomato (2C = 1.96 pg) as an external reference standard, the 2C DNA content was determined as 1.810 ± 0.008 and 1.813 ± 0.004 for the leaves and the callus, respectively, indicating that the 2C DNA content and ploidy status remained unchanged in the callus when compared to the donor plant (Figure 3).

### 2.4. Phytochemical Analysis

#### 2.4.1. Phenol and Flavonoid Contents

**Figure 4:**
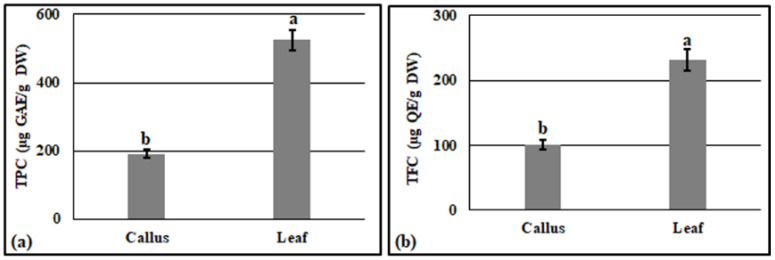
Total phenolic content expressed as gallic acid equivalent (**a**) and total flavonoid content expressed as quercetin equivalent (**b**). Values are the mean of three replicates ± S.D. Letters a and b on bars indicate the significant differences according to Duncan’s test (*p* < 0.05).

**Figure 5:**
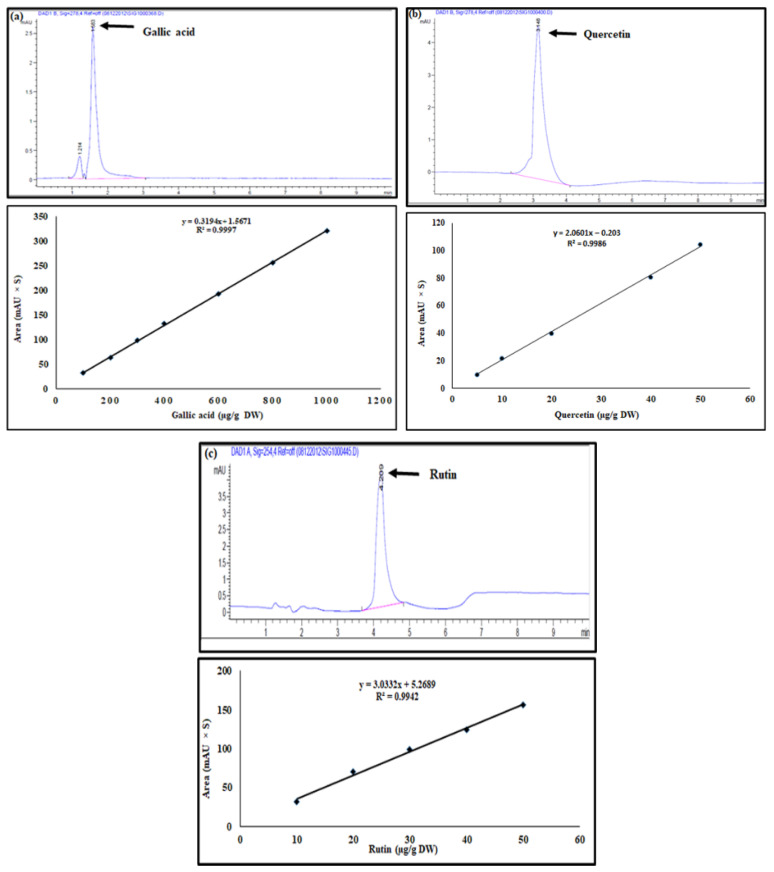
HPLC chromatogram of the standards (**a**) gallic acid, (**b**) quercetin, and (**c**) rutin and their calibration plots.

The screenings of the total phenolic content and the total flavonoid content in the methanol extracts of the leaves and callus were firstly performed using a UV spectrophotometer. The determination of total phenols was calculated based on gallic acid (GA), while the quercetin was used for the total flavonoid estimation. The results showed that the phenolic content was higher than the total flavonoids in both the callus and leaf extracts. Moreover, the lowest total phenol content (191 µg GAE/g DW) and total flavonoids (100 µg QE/g DW) were detected in the callus compared to the leaf extracts (Figure 4). On the other hand, the gallic acid, quercetin, and rutin (Figure 5) were observed to have a significant difference between the callus and the leaf extracts when they were quantified using HPLC. Indeed, for the leaf extracts, the recorded values of the gallic acid, quercetin, and rutin were 2.7, 1.5, and 1.7-times more, respectively, than the values detected in the callus extracts (Table 1).

#### 2.4.2. GC-MS Analysis

An analysis was performed to determine the bioactive compounds present in the methanolic extracts of the leaves and callus of *A. precatorious* using gas chromatography and mass spectroscopy (GC-MS). The results presented in Table 2 and Figure S1 showed the detection of 22 phytochemical compounds in the callus extracts, while 28 phytochemical compounds were found in the leaf extract (Table 2, Figure S2). Based on the peak area percentage, the three major compounds detected in the callus extract were n-hexadecanoic acid (17.22%); 2-undecenal (11.82%); and 2-decenal, (E)- (10.57%). However, octadecanoic acid; 2-propenyl ester; 2-undecenal; and 2-decenal, (E)- were the top three major phytocompunds found in the leaf extract, with percentages of 21.77%, 11.19%, and 10.14%, respectively. On the other hand, a variation was noticed when comparing the types of compounds obtained from the callus and leaves. For instance, tridecanal; heneicosane; 9-octadecenoic acid; methyl ester, (E)-; 2(3H)-furanone, 5-dodecyldihydro-; pentacosane; tetracontane; and tetratriacontane were detected only in the callus, whereas furan, 2-butyltetrahydro, 3-hexanone, 2-hexanone, 3-hexanol, 2-hexanol, octanoic acid, nonane, 2-methyl-5-propyl-, vinyl caprylate, 2-dodecenal, decanoic acid, 2-propenyl ester, and bis(2-ethylhexyl) phthalate were revealed in the leaf extract and absent in the callus extract.

## 3. Discussion

In the current work, the genome and phytochemical compounds in the leaves and callus of the *A. precatorius* were estimated. At the beginning, the plant species was identified by the amplification of the internal transcribed spacer (ITS) region, as this region is an important molecular target to identify species due to the sequence variation that exists within them and the speed at which these sequences evolve [48]. The internal transcribed spacer (ITS) region is the most commonly sequenced locus for plant molecular systematic investigations at the species level. Furthermore, it has been noted that ITS reduces species-level variability in certain groups and can be amplified in two smaller fragments (ITS1 and ITS2) adjoining the 5.8S locus, which is useful for degraded samples [49].The success rate for the identification of the collected plant as *A. precatorius* was more than 97% using the BLAST method. The high sequencing success rate of the ITS in the identification of plants has been noted in different plant species [50,51]. 

Because of their hard seed coat, the propagation of *A. precatorius* is difficult. Thus, the development of this medicinal plant through in vitro propagation is a requirement to save it from a further decrease [11]. In this study, an *A. precatorius* stem-derived callus was successfully induced on MS medium with several combinations of BA and 2,4-D: the most effective combination was 0.5 µM BA and 5 µM 2,4-D, which produced the callus with the highest fresh weight compared to the other treatments (Figure 2). These findings are consistent with a previous study [52] which noted that Murashige and Skoog culture medium containing 2,4-dichlorophenoxyacetic acid in combination with 6-benzylaminopurine is most suitable for the induction of a friable morphogenic callus in a grain legume, the *Cyamopsis tetragonoloba* plant. Similarly, for the lotus *Nelumbo nucifera*, the highest efficiency of callus production was induced in the explants grown on Murashige and Skoog (MS) basal medium containing an optimized combination of 2,4-dichlorophenoxyacetic acid (2,4-D) and 6-benzylaminopurine (6-BA) [53]. In wheat plants, 2,4-D alone or in combination with cytokinins has been used for the induction of callus initiation [54]. However, the explant type and plant genotypes were previously reported to be key factors affecting callus induction. The PGRs also play a crucial role in in vitro morphogenesis, for example in callus induction, and the combinations of PGRs needed in plant tissue culture may differ among plant species.

During in vitro propagation, the chemical composition of the culture medium and the presence of growth regulators often promotes genetic disturbances, which result in somaclonal variation. This can be the result of chromosomal aberrations or changes in the number of chromosomes or ploidy level. Therefore, genome stability control is desirable for growing plants in vitro. As a fast and accurate method for the estimation of nuclear DNA content, flow cytometry has been successfully used to study genome changes in micropropagated plants [55]. In this study, we noticed that the analyzed donor *A. precatorius* and the in vitro regenerated callus were diploid (Figure 3). Moreover, no variation in genome size could be linked to the chemical composition and the presence of growth regulators in the culture medium. The estimated 2C DNA content in the donor pant and the callus were 1.810 ± 0.008 and 1.813 ± 0.004 pg, respectively and, therefore, approximately 1.77 × 10^9^ and 1.773 × 10^9^ bp, respectively. In agreement with our findings., the production of genetically-stable plants was previously reported in *Rauvolfia serpentine* [12], *Asclepias latifolia*, *A. speciosa* and *A. subverticillata*, [56], and *Viola uliginosa* [57]. In contrast, the authors of [58] and [59] observed a variation of nuclear DNA content in in vitro regenerated *Elaeis guineensis* and *Plantago asiatica* L plants. Additionally, in a wide range of crops, prolonged callus phase regeneration can result in an increase in the ploidy instability rate [60].

In vitro regeneration has been widely used to avoid destroying the natural habitat by harvesting the roots and the underground parts of the plants. For instance, industries use in vitro regeneration to produce bioactive compounds from medicinal plants to ensure a continuous supply in a relatively short period of time [61]. Phenolic and flavonoid compounds are commonly known as plant secondary metabolites. Further, these metabolites are used in herbal medicines and have been reported to be interesting candidates for pharmaceutical and medical applications, as they have antioxidant, anticancer, and antimicrobial properties [62]. Previously, it was noted that the callus has a higher total phenolic and flavonoid than wild explants [63]. Callus culture is very useful to obtain commercially important secondary metabolites, especially when it is regenerated by the addition of NAA in the culture medium. The presence of NAA induces a high stress level which leads to an accumulation of more phenolic in the produced callus [64]. In our study, the content of total phenols and flavonoids in the leaves was higher than that obtained in the callus (Figure 4). Moreover, in both the callus and leaves, the phenolic compounds were more abundant than the flavonoids. This is in agreement with the findings of the authors of [65], who noted that a greater total phenolic content (25.48 ± 0.62 GAE mg/g) was found in *A. precatorius* leaves extracts compared to the total flavonoid content (17.16 ± 1.04 QE mg/g). On the other hand, the quantification by HPLC showed that the gallic acid, quercetin, and the rutin contents in the callus were less than that found in the leaves (Table 1). Similarly, the authors of [66] found that the total phenolic compounds and flavonoid content of *Phyllanthus fraternus* stems were higher than in the stems of induced callus.

The total chemical constituents were screened by the use of GC-MS; Table 2 summarizes the chemical compounds detected in the callus and leaf extracts, as well as their biological activities. Based on the peak area, the most dominant compound in the callus extract was n-hexadecanoic acid (17.22%), which possesses several biological activities, such as anti-inflammatory, antioxidant, nematicide, and pesticide activities [34,67]. However, octadecanoic acid, 2-propenyl ester was the most prevailing compound (21.77%) in the lead extract; it has antibacterial and anti-inflammatory activities [36,67]. Previously, 13 compounds were identified in the petroleum ether leaf extract of *A. precatorius*; out of these, two (octadecanoic acid and n-hexadecanoic acid) were detected in our extracts. According to [8], these compounds are considered as potential phyto-compounds to target the calcitonin gene-related peptide protein to treat migraine headaches. Additionally, the authors of [10] showed the presence of various classes of secondary metabolites, such as alkaloids, flavonoids, phenols, tannins, and steroids, in *A. precatorius* leaf extract.

## 4. Materials and Methods

### 4.1. Plant Material Collection

**Figure 6:**
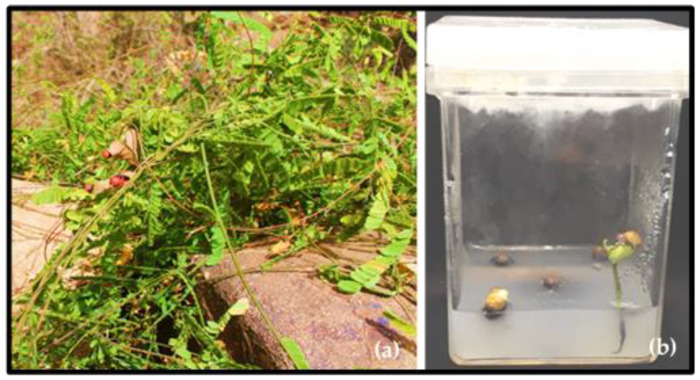
(**a**) Photos of *Abrus precatorius* collected from the Al-Baha mountains, Saudi Arabia. (**b**) the germination of the seeds.

The pods containing seeds of the endangered species *A. precatorius* were collected from the Al-Baha mountains, Saudi Arabia and brought to the lab in the Department of Botany and Microbiology, College of Science, King Saud University. The seeds were surface sterilized in 10% (*v*/*v*) commercial Clorox bleach for 20 min and then rinsed three times with sterile deionized water. Afterwards, the seeds were transferred to jars containing 3% agar and 2% sucrose (five seeds per jar) for germination (Figure 6). Once the seeds were germinated, seedlings were transplanted into plastic pots containing soil (1/3) and peat moss (2/3) and irrigated with half-strength Hoagland nutrient solution [68]. The pots were maintained in the growth chamber under controlled conditions. Two months after planting, the stems were used as explant sources for the callus induction and the leaves were harvested for further use.

### 4.2. Callus Induction

The stems were excised from two-month-old seedlings and were used for callus induction. The explants were firstly washed under running tap water, surface sterilized with 10% beach for 15 min, washed several times with sterilized double-distilled water, and were finally cultured on solidified (0.7% agar) MS medium [69] supplemented with 0.5, 1, 2.5, and 5 µM of 6-Benzylaminopurine (BAP) and 2,4-dichlorophenoxyacetic acid (2,4-D) in different combinations and enriched with 30 g/L sucrose. One month later, the vigorous callus growth was noted by measuring the fresh weight and the best callus was selected for the genome estimation and the identification of phytochemical compounds.

### 4.3. DNA Extraction and ITS Identification

The DNA was isolated from leaf samples from two-month-old seedlings using a DNeasy Plant Mini Kit (Qiagen, Hilden, Germany), following the manufacturer’s instructions. The DNA quantity was measured using a Nanodrop 8000 spectrophotometer (Thermo Scientific, Wilmington, NC, USA) and the integrity was checked with 1% agarose gel electrophoresis. Then, the ITS region was amplified using the universal primers ITS1 (forward) and ITS4 (reverse), as described in [70]. The PCR amplification was carried out in a 25 µL reaction volume containing PuReTaq^TM^ Ready-To-Go^TM^ PCR beads (GE Healthcare, Little Chalfont, Buckinghamshire, UK).), 0.5 µL of each primer, 2 µL of DNA, and 20 µL of water. The PCR reaction was performed in an Applied Biosystems thermal cycler (Applied Biosystems, Waltham, MA, USA), using the following conditions: one cycle at 94 °C for 5 min, 30 cycles at 94 °C for 1 min, annealing at 50 °C for 1 min, extension at 72 °C for 1 min, and a final extension step of 72 °C for 5 min. The PCR product was examined on a 1.2% agarose gel and then sent to Macrogen Inc. (Geumchun-gu, Seoul, Korea)) for bidirectionally sequencing. The obtained sequence was subjected to a BLAST search in the NCBI databases to identify the plant species.

### 4.4. Genome Size Estimation

#### 4.4.1. Nuclei Extraction

All steps of nuclei extraction were performed on ice (4 °C) and the materials used in the experiment were cleaned thoroughly. For the nuclei isolation, the buffer was prepared as described in [15], with 45 mm MgCl_2_, 20 mm MOPS, 30 mm sodium citrate, and 0.1% Triton X-100 (*v*/*v*), at a pH 7.4 supplemented by 1% PVP and 0.5% β-mercaptoethanol, which were freshly added for the extraction of pure nuclei.

The young leaves and the callus of the best phytohormone (BA and 2,4-D) combinations (20 mg) were chopped with a sharp razor blade into 0.4–0.8 mm size on a sterile Petri plate containing 700 µL of cold Gailbraith buffer. Then, the suspension was mixed by pipetting and filtered through a 20 µm double nylon mesh. Afterwards, the nuclei suspension was stained for 12 min with 50 µg/mL of PI (propidium iodide, Sigma, St. Louis, MO, USA) and samples were stored on ice prior to analysis.

#### 4.4.2. Flow Cytometric Analysis

Nuclear DNA content in the sample was calculated according to [71]. The fluorescence of a minimum of 5000 propidium iodide-stained nuclei was estimated using a Muse Cell Analyzer flow cytometer (Merck Millipore, Burlington, MA, USA). The flow rate of the capillary was set 0.12 µL/s, which is very low. The external reference, *Solanum lycopersicum*’ (2C = 1.96 pg), was kindly provided by Dr. Jaroslav Dolezel, Laboratory of Molecular Cytogenetics and Cytometry, Institute of Experimental Botany, Czech Republic. Propidium iodide was measured at 585 nm to read the 2C nuclei DNA content of the sample. The obtained histograms were computerized using the Muse Cell Analyzer software packages (Muse 1.8 analyses, Burlington, MA, USA). The samples’ 2C DNA content was calculated according to the following formula:

$$Abrus\;precatorious\;\text{2C DNA content}\;=\frac{\text{Florescence mean intensity of}\;A\text{.}\;precatorious}{\text{Floresence mean intensity of standrad}}\;\times \;\text{2C DNA content of standard}$$

The number of base pairs per haploid genome was calculated based on the equivalent of 1 pg DNA = 965 mega base pairs [72].

### 4.5. Total Phenolic and Total Flavonoid Content

#### 4.5.1. Extract Preparation

The leaves and the callus were dried at room temperature then grinded using a TissueLyser LT machine (Qiagen, Hilden, Germany ), and 2 g of the dry powder was extracted by shaking for 24 h in 20 mL of 80% methanol. Thereafter, the extracted solutions were centrifuged at 5000 rpm for 5 min and the supernatants were evaporated. Finally, the extracts were dissolved and filtered through a 0.45 µm membrane and were stored at 4 °C until the time of analysis.

#### 4.5.2. Total Phenolic Content Estimation

The total phenolic content was estimated using the Folin–Ciocalteu colorimetric method as described in [73]. A total of 0.5 mL of the diluted extracts were mixed thoroughly with 0.125 mL of the Folin–Ciocalteu reagent, left to react for 5 min, then 1.25 mL of 7% sodium carbonate was added to the mixture. The volume was adjusted to 3 mL by adding water. After incubation for 90 min at room temperature, the OD of the reaction mixtures was measured at a 760 nm wavelength using a UV–1800 spectrophotometer (Shimadzu UV spectrophotometer). Total phenolic content was expressed as microgram gallic acid equivalents per gram dry weight through the calibration curve of gallic acid with the equation (y = 0.0056x + 0.0542, R^2^ = 0.9908).

#### 4.5.3. Total Flavonoid Content Estimation

The total flavonoid concentration was estimated using the calorimetric method, as described in [74]. Briefly, 0.5 mL of the sample extract was added to an equal volume of 2% AlCl_3_ and then the mixture was incubated for 1 h at the room temperature. The absorbance was determined at 420 nm, and the flavonoid content was measured as microgram quercetin equivalent per gram dry weight (µg QE g^−1^ DW) from the calibration curve developed for the quercetin.

### 4.6. HPLC Quantification of Gallic Acid, Quercetin, and Rutin

The extracts and the standards (gallic acid, quercetin, and rutin) were dissolved in methanol. The mobile phases used for the separation and quantification consisted of the following: 1% acetic acid solution (A) and methanol (B) (40:60) (*v*/*v*) for the gallic acid, and acetonitrile (A) and methanol (B) (50:50) (*v*/*v*) for the quercetin and rutin. The HPLC analysis was carried out using an Agilent Technologies 1290 Infinity system. The separations were performed in a ZOBRAX RX-C18 column (4.6 × 150 mm) in which the mobile phases were pumped at a flow rate of 1 ml/ min, with a run time of 10 min and injection volume of 10 μL at a temperature of 25 °C. The gallic acid, quercetin, and rutin of the callus and leaf extracts were estimated by referencing them to the calibration curves prepared from their authentic standards.

### 4.7. Gas Chromatography Mass Spectrometry Analysis

Organic phases were analyzed by GC-MS (QP2010 Ultra, Shimadzu, Tokyo, Japan). An Rtx-5MS column (30 m; 0.25 mm i.d.; 0.25 µm) was used. Helium was employed as the carrier gas at a constant column flow of 1.6 mL/min. The GC oven temperature program was as follows: 50 °C for 3 min, increased at 10 °C/min to 280 °C, hold for 3 min, increased at 2 °C/min to 300 °C, and then maintained at this temperature for 10 min. The injector and detector temperatures were held at 250 and 275 °C, respectively. The identification of the organic phase was performed by MS running in scan mode at 70 eV and using electron impact ionization. The mass spectra obtained were compared with those in the NIST library database.

### 4.8. Statistical Analysis

The statistical analysis was performed using the software IBM-SPSS version 26. The data of three replicates were subjected to one-way ANOVA and analyzed with Duncan’s test. Different letters were used to indicate the significant difference at *p* ≤ 0.05.

For the identification of *Abrus precatorius*, the obtained sequences were compared by the BLAST algorithm and a phylogenetic tree was constructed using MEGA X software and the neighbor-joining method by bootstrapping 1000 times.

## 5. Conclusions

In the present study, the leaves of *A. precatorius* and the induced callus were compared for their genome size and phytochemical constituents. BA and 2,4-D in concentrations of 0.5 µM and 5 µM, respectively, were the best combination for callus induction and growth. Analysis using flow cytometry proved that the induced callus approximately maintained the same genomic DNA content as the donor plant. On the other hand, we noted that the total phenolic compounds and flavonoid content of leaves were higher than in the callus. Finally, the GC-MS analysis confirmed that callus cultures can be source of high-value secondary metabolites that possess various biological activities. Based on the peak area, the most dominant compounds in the callus and the leaf extracts were n-hexadecanoic acid (17.22%) and octadecanoic acid, 2-propenyl ester (21.77%), respectively. Therefore, biological and pharmacological studies are recommended to appraise these metabolites.

## Figures and Tables

**Table 1:** Gallic acid, quercetin, and rutin content (µg/g DW) in the leaf and callus extracts.

Plant Material	Gallic Acid	Quercetin	Rutin
Leaves	180.77 ± 6.17	73.40854 ± 3.58	24.13197 ± 1.26
Callus	65.7061 ± 5.58	48.32039 ± 4.55	14.39261 ± 1.33

**Table 2:** Phytocompounds of callus and leaf extracts identified by GC-MS analysis.

Callus Extract				Leaf Extract			
Compounds	RT	%	Bioactivity	Compounds	RT	%	Bioactivity
Decanal	11.00	2.81	Antioxidant, antimicrobial, and antitumor [18]	Furan, 2-butyltetrahydro	3.125	0.78	Chemopreventive properties [19]
2-Decenal, (E)-	11.89	10.57	Nematicidal activity [20]	3-Hexanone	3.309	2.08	Antimicrobial [21]
Nonanoic acid	12.018	2.42	Antimicrobial [22]	2-Hexanone	3.396	2.65	Antibacterial activity [23]
Undecanal	12.567	1.73	Antimicrobial and antioxidant activities [24]	3-Hexanol	3.396	0.68	Antimicrobial [25]
2-Undecenal	13.414	11.82	Antimicrobial and antioxidant activities [24]	2-Hexanol	3.587	1.76	Antimicrobial [26]
Dodecanal	14.029	2.35	Antimicrobial [27]	Octanoic acid	10.571	4.04	Tremor suppressing [28]
Tridecanal	15.396	1.72	Antimicrobial [29]	Decanal	11.015	2.18	Antioxidant, antimicrobial, and antitumor [18]
Tetradecanal	16.679	1.57	Antioxidant and antibacterial activities [30]	2-Decenal, (E)-	11.914	10.14	Nematicidal activity [20]
Pentadecanal-	17.896	1.78	Antibacterial [31]	Nonanoic acid	12.058	1.69	Antimicrobial [22]
Heneicosane	18.805	3.44	Antimicrobial [32]	Undecanal	12.569	1.44	Antimicrobial and antioxidant activities [24]
Heneicosane	19.889	2.46	Antimicrobial [32]	2-Undecenal	13.213	1.25	Antimicrobial and antioxidant activities [24]
Hexadecanoic acid, methyl ester	20.205	8.35	Antibacterial activities [33]	2-Undecenal	13.425	11.19	Antimicrobial and antioxidant activities [24]
n-Hexadecanoic acid	20.613	17.22	Anti-inflammatory [34]	Nonane, 2-methyl-5-propyl-	13.835	1.05	Anti-cancer [35]
Octadecanoic acid, 2-propenyl ester	21.721	7.16	Antibacterial [36]	Dodecanal	14.028	2.03	Antimicrobial [27]
9-Octadecenoic acid, methyl ester, (E)-	21.987	6.99	Antimicrobial and nematicidal [37]	Vinyl caprylate	14.166	0.71	Antimicrobial [38]
2(3H)-Furanone, 5-dodecyldihydro-	22.105	2.38	Antibacterial [39]	Trifluoroacetic acid, n-tridecyl ester	14.568	0.75	Antibacterial, antifungal [40]
Palmitic acid vinyl ester	23.157	3.00	Antimicrobial activity [41]	2-Dodecenal	14.837	1.01	Nematicidal activity [20]
Octadecanoic acid, 2-propenyl ester	23.591	3.30	Antibacterial [36]	Pentadecane	15.184	0.78	Antimicrobial and antioxidant activity [42]
Heneicosane	23.753	1.68	Antimicrobial activity [32]	Tetradecanal	15.389	1.47	Antioxidant and antibacterial activities [30]
Pentacosane	24.624	2.80	Antimicrobial activity [43]	Tetradecanal	16.672	1.64	Antioxidant and antibacterial activities [30]
Tetracontane	25.459	2.84	Antioxidant and antimicrobial activity [44]	Decanoic acid, 2-propenyl ester	17.416	0.97	Antimicrobial activity [45]
Tetratriacontane	26.269	1.61	Antibacterial and antifungal [46]	Pentadecanal	17.886	2.17	Antibacterial (Ricciardelli, et al., 2020)
				Hexadecanoic acid, methyl ester	20.195	2.74	Antibacterial activities [33]
				n-Hexadecanoic acid	20.615	1.28	Anti-inflammatory [34]
				Octadecanoic acid, 2-propenyl ester	21.711	21.77	Antibacterial [36]
				Palmitic acid vinyl ester	23.151	8.83	Antimicrobial activity [41]
				Octadecanoic acid, 2-propenyl ester	23.579	10.60	Antibacterial [36]
				Bis(2-ethylhexyl) phthalate	25.985	2.33	Antimicrobial and cytotoxic activity [47]

## Data Availability

Data are contained within the article or supplementary material.

## Supplementary Materials

The following are available online at https://www.mdpi.com/article/10.3390/plants11040567/s1, Figure S1: GC-MS chromatogram of the bioactive compounds present in the callus extract, Figure S2: GC-MS chromatogram of the bioactive compounds present the leaves extract.

## References

[1] Rahman M.A., Mossa J.S., Al-Said M.S., Al-Yahya M.A. (2004). Medicinal plant diversity in the flora of Saudi Arabia 1: A report on seven plant families. Fitoterapia.

[2] Al-Khulaidi A., Al-Sagheer N.A., Al-Turki T., Filimban F. (2018). Inventory of most rare and endangered plant species in Albaha region Saudi Arabia. Ijbpas.

[3] Thomas J., El-Sheikh M.A., Alatar A.A. (2017). Endemics and endangered species in the biodiversity hotspot of the Shada Mountains, Saudi Arabia. J. Arid. Land.

[4] Al-Asmari A.K., Abbasmanthiri R., Osman N.M.A., Al-Asmari B.A. (2020). Endangered Saudi Arabian plants having ethnobotanical evidence as antidotes for scorpion envenoming. Clin. Phytosci..

[5] Abbas A.M., Al-Kahtani M.A., Alfaifi M.Y., Elbehairi S.E.I., Badry M.O. (2020). Floristic diversity and phytogeography of Jabal Fayfa: A subtropical dry zone, south-west Saudi Arabia. Diversity.

[6] Garaniya N., Bapodra A. (2014). Ethno botanical and Phytophrmacological potential of *Abrus precatorius* L.: A review. Asian Pac. J. Trop. Biomed..

[7] Bhatia M., Siddiqui N., Gupta S. (2013). *Abrus precatorius* (L.): An evaluation of traditional herb. J. Pharm. Res..

[8] Parthasarathy V., TV A.K. (2019). Screening of potential GCMS derived antimigraine compound from the leaves of *Abrus precatorius* Linn to target “calcitonin gene related peptide” receptor using in silico analysis. Food Sci. Hum. Wellness.

[9] Wan-Ibrahim W.S., Tuan Ismail T.N.N., Mohd-Salleh S.F., Ismail N. (2018). GC-MS analysis of phytochemical compounds in aqueous leaf extract of *Abrus precatorius*. Pertanika J. Trop. Agric. Sci..

[10] Pavithra K., Uddandrao V.S., Mathavan S., Gobeeswaran N., Vadivukkarasi S., Ganapathy S. (2020). Identification of bioactive factors from *Abrus precatorius* by GC–MS, NMR and evaluation of its antioxidant activity. Mater. Today: Proc..

[11] Biswas A., Roy M., Miah M.B., Bhadra S. (2007). In vitro propagation of *Abrus precatorius* L.-a rare medicinal plant of Chittagong hill tracts. Plant Tissue Cult. Biotechnol..

[12] Zafar N., Mujib A., Ali M., Tonk D., Gulzar B., Malik M., Sayeed R., Mamgain J. (2019). Genome size analysis of field grown and tissue culture regenerated *Rauvolfia serpentina* (L.) by flow cytometry: Histology and scanning electron microscopic study for in vitro morphogenesis. Ind. Crops Prod..

[13] Thiem B., Śliwińska E. (2003). Flow cytometric analysis of nuclear DNA content in cloudberry (*Rubus chamaemorus* L.) in vitro cultures. Plant Sci..

[14] Orzechowska M., Stępień K., Kamińska T., Siwińska D. (2013). Chromosome variations in regenerants of Arabidopsis thaliana derived from 2-and 6-week-old callus detected using flow cytometry and FISH analyses. Plant Cell Tissue Organ Cult. (PCTOC).

[15] Galbraith D.W. (1989). Analysis of higher plants by flow cytometry and cell sorting. Int. Rev. Cytol..

[16] Bourge M., Brown S.C., Siljak-Yakovlev S. (2018). Flow cytometry as tool in plant sciences, with emphasis on genome size and ploidy level assessment. Genet. Appl..

[17] Pellicer J., Powell R.F., Leitch I.J. (2021). The application of flow cytometry for estimating genome size, ploidy level endopolyploidy, and reproductive modes in plants. Molecular Plant Taxonomy.

[18] Liu K., Chen Q., Liu Y., Zhou X., Wang X. (2012). Isolation and biological activities of decanal, linalool, valencene, and octanal from sweet orange oil. J. Food Sci..

[19] Nisha S.R., Jeeva S., Paul Raj K. (2018). Chemical profiling and compound Isolation of different extracts of Boucerosia pauciflora Wight using GCMS Analysis. J. Emerg. Technol. Innov. Res..

[20] Caboni P., Ntalli N.G., Aissani N., Cavoski I., Angioni A. (2012). Nematicidal activity of (E, E)-2, 4-decadienal and (E)-2-decenal from Ailanthus altissima against Meloidogyne javanica. J. Agric. Food Chem..

[21] Kawuri R., Darmayasa I. (2019). Bioactive compound from extract filtrat streptomyces sp. Sp1. as biocontrol of vibriosis on larvae of Macrobrachium rosenbergii shrimps. Hayati J. Biosci..

[22] Sahin N., Kula I., Erdogan Y. (2006). Investigation of antimicrobial activities of nonanoic acid derivatives. Fresenius Environ. Bull..

[23] PATIL V.-I.R., Donde K.J., Jadhav S.B., Malve S.P. (2002). Synthesis and antimicrobial activity of 3-hydroxyimino-5-methyl-2-hexanone (himh) and its dioxime derivative. Acta Pol. Pharm..

[24] Yildiz H. (2016). Chemical composition, antimicrobial, and antioxidant activities of essential oil and ethanol extract of *Coriandrum sativum* L. leaves from Turkey. Int. J. Food Prop..

[25] Jenecius A., Uthayakumaria F., Mohan V. (2012). GC-MS determination of bioactive components of *Sauropus bacciformis* blume (Euphorbiaceae). J. Curr. Chem. Pharm. Sci..

[26] Kumar Tyagi A., Bukvicki D., Gottardi D., Veljic M., Guerzoni M.E., Malik A., Marin P.D. (2013). Antimicrobial potential and chemical characterization of serbian liverwort (*Porella arboris-vitae*): SEM and TEM observations. Evid.-Based Complement. Altern. Med..

[27] Chanprapai P., Kubo I., Chavasiri W. (2018). Anti-rice pathogenic microbial activity of *Persicaria* sp. Extracts. Sci. Technol. Asia.

[28] Nahab F.B., Handforth A., Brown T., Shin C., Quesada A., Dong C., Haubenberger D., Hallett M. (2012). Octanoic acid suppresses harmaline-induced tremor in mouse model of essential tremor. Neurotherapeutics.

[29] Yücel T.B., Karaoğlu Ş.A., Yaylı N. (2017). Antimicrobial Activity and Composition of Rindera lanata (LAM.) Bunge var. canescens (ADC) Kosn. Essential oil Obtained by Hydrodistillation and Microwave Assisted Distillation. Rec. Nat. Prod..

[30] Bittencourt M.L., Ribeiro P.R., Franco R.L., Hilhorst H.W., de Castro R.D., Fernandez L.G. (2015). Metabolite profiling, antioxidant and antibacterial activities of Brazilian propolis: Use of correlation and multivariate analyses to identify potential bioactive compounds. Food Res. Int..

[31] Ricciardelli A., Casillo A., Corsaro M.M., Tutino M.L., Parrilli E., van der Mei H.C. (2020). Pentadecanal and pentadecanoic acid coatings reduce biofilm formation of Staphylococcus epidermidis on PDMS. Pathog. Dis..

[32] Vanitha V., Vijayakumar S., Nilavukkarasi M., Punitha V., Vidhya E., Praseetha P. (2020). Heneicosane—A novel microbicidal bioactive alkane identified from *Plumbago zeylanica* L.. Ind. Crops Prod..

[33] Shaaban M.T., Ghaly M.F., Fahmi S.M. (2021). Antibacterial activities of hexadecanoic acid methyl ester and green-synthesized silver nanoparticles against multidrug-resistant bacteria. J. Basic Microbiol..

[34] Aparna V., Dileep K.V., Mandal P.K., Karthe P., Sadasivan C., Haridas M. (2012). Anti-inflammatory property of n-hexadecanoic acid: Structural evidence and kinetic assessment. Chem. Biol. Drug Des..

[35] Jenifer D.R., Malathy B., SS A. (2021). In vitro and in silico studies on the biochemistry and anti-cancer activity of phytochemicals from *Plumb. Zeylanica*. Indian J. Biochem. Biophys. (IJBB).

[36] Hanafy S.M., Abd El-Shafea Y.M., Saleh W.D., Fathy H.M. (2021). Chemical profiling, in vitro antimicrobial and antioxidant activities of pomegranate, orange and banana peel-extracts against pathogenic microorganisms. J. Genet. Eng. Biotechnol..

[37] Ali A., Javaid A., Shoaib A. (2017). GC-MS analysis and antifungal activity of methanolic root extract of Chenopodium album against Sclerotium rolfsii. Planta Daninha.

[38] Park S.J., Sin Y.M., Lee S.O., Lee T.H. (2002). Enzymatic synthesis of an acylated phloroglucinol derivative with phloroglucinol and vinyl octanoate in acetonitrile. Biotechnol. Lett..

[39] Yue X.-F., Shang X., Zhang Z.-J., Zhang Y.-N. (2017). Phytochemical composition and antibacterial activity of the essential oils from different parts of sea buckthorn (*Hippophae rhamnoides* L.). J. Food Drug Anal..

[40] Kelechi Oleru A.O., Olayiwola J., Popoola B. (2021). Potential Antimicrobial Substances from the Characterized Bioactive Compounds Extracted from Secondary Metabolites of Aspergillus terreus. Res. J. Microbiol..

[41] Uma B., Prabhakar K., Rajendran S., Lakshmi S.Y. (2009). Studies on GC/MS spectroscopic analysis of some bioactive antimicrobial compounds from *Cinnamomum zeylanicum*. J. Med. Plants.

[42] Begum I.F., Mohankumar R., Jeevan M., Ramani K. (2016). GC–MS analysis of bio-active molecules derived from *Paracoccus pantotrophus* FMR19 and the antimicrobial activity against bacterial pathogens and MDROs. Indian J. Microbiol..

[43] Marrufo T., Nazzaro F., Mancini E., Fratianni F., Coppola R., De Martino L., Agostinho A.B., De Feo V. (2013). Chemical composition and biological activity of the essential oil from leaves of *Moringa oleifera* Lam. cultivated in Mozambique. Molecules.

[44] Rhetso T., Shubharani R., Roopa M., Sivaram V. (2020). Chemical constituents, antioxidant, and antimicrobial activity of *Allium chinense* G. Don. Future J. Pharm. Sci..

[45] Bharath M., Azeem M., Basha S., Keerthan H. (2016). Antimicrobial Activity of Cinnamon Extracts against Foodborne Pathogens *E. coli*, *S. tyhimurium* and *S. aureus* and *L. monocytogens*. Curr. Nutr. Food Sci..

[46] Abubakar M.N., Majinda R.R. (2016). GC-MS analysis and preliminary antimicrobial activity of *Albizia adianthifolia* (Schumach) and *Pterocarpus angolensis* (DC). Medicines.

[47] Lotfy M.M., Hassan H.M., Hetta M.H., El-Gendy A.O., Mohammed R. (2018). Di-(2-ethylhexyl) Phthalate, a major bioactive metabolite with antimicrobial and cytotoxic activity isolated from River Nile derived fungus *Aspergillus awamori*. Beni-Suef Univ. J. Basic Appl. Sci..

[48] Raja H.A., Miller A.N., Pearce C.J., Oberlies N.H. (2017). Fungal identification using molecular tools: A primer for the natural products research community. J. Nat. Prod..

[49] Kress W.J., Wurdack K.J., Zimmer E.A., Weigt L.A., Janzen D.H. (2005). Use of DNA barcodes to identify flowering plants. Proc. Natl. Acad. Sci. USA.

[50] Pathak M.R., Mohamed A.A., Farooq M. (2018). DNA barcoding and identification of medicinal plants in the kingdom of Bahrain. Am. J. Plant Sci..

[51] Zhao L.L., Feng S.J., Tian J.Y., Wei A.Z., Yang T.X. (2018). Internal transcribed spacer 2 (ITS 2) barcodes: A useful tool for identifying *Chinese Zanthoxylum*. Appl. Plant Sci..

[52] Prem D., Singh S., Gupta P.P., Singh J., Kadyan S.P.S. (2005). Callus induction and de novo regeneration from callus in Guar (*Cyamopsis tetragonoloba*). Plant Cell Tissue Organ Cult..

[53] Deng X., Xiong Y., Li J., Yang D., Liu J., Sun H., Song H., Wang Y., Ma J., Liu Y. (2020). The Establishment of an Efficient Callus Induction System for Lotus (*Nelumbo nucifera*). Plants.

[54] Malik S.I., Rashid H., Yasmin T., Minhas N.M. (2003). Effect of 2, 4-dichlorophenoxyacetic acid on callus induction from mature wheat (*Triticum aestivum* L.) seeds. Int. J. Agric. Biol..

[55] Sliwinska E., Thiem B. (2007). Genome size stability in six medicinal plant species propagated in vitro. Biol. Plant..

[56] Sakhanokho H.F., Babiker E.M., Smith B.J., Drackett P.R. (2019). High-frequency somatic embryogenesis, nuclear DNA estimation of milkweed species (*Asclepias latifolia*, *A. speciosa*, and *A. subverticillata*), and genome size stability of regenerants. Plant Cell Tissue Organ Cult. (PCTOC).

[57] Slazak B., Sliwinska E., Saługa M., Ronikier M., Bujak J., Słomka A., Göransson U., Kuta E. (2015). Micropropagation of *Viola uliginosa* (Violaceae) for endangered species conservation and for somaclonal variation-enhanced cyclotide biosynthesis. Plant Cell Tissue Organ Cult. (PCTOC).

[58] Lucia G., Castiglione M.R., Turrini A., Ronchi V.N., Geri C. (2011). Cytogenetic and histological approach for early detection of “mantled” somaclonal variants of oil palm regenerated by somatic embryogenesis: First results on the characterization of regeneration system. Caryologia.

[59] Makowczyńska J., Andrzejewska-Golec E., Sliwinska E. (2008). Nuclear DNA content in different plant materials of *Plantago asiatica* L. cultured in vitro. Plant Cell Tissue Organ Cult..

[60] Borchert T., Fuchs J., Winkelmann T., Hohe A. (2007). Variable DNA content of Cyclamen persicum regenerated via somatic embryogenesis: Rethinking the concept of long-term callus and suspension cultures. Plant Cell Tissue Organ Cult..

[61] Park J.-S., Seong Z.-K., Kim M.-S., Ha J.-H., Moon K.-B., Lee H.-J., Lee H.-K., Jeon J.-H., Park S.U., Kim H.-S. (2020). Production of flavonoids in callus cultures of *Sophora flavescens* Aiton. Plants.

[62] Tungmunnithum D., Thongboonyou A., Pholboon A., Yangsabai A. (2018). Flavonoids and other phenolic compounds from medicinal plants for pharmaceutical and medical aspects: An overview. Medicines.

[63] Rameshkumar R., Satish L., Pandian S., Rathinapriya P., Rency A.S., Shanmugaraj G., Pandian S.K., Leung D.W., Ramesh M. (2018). Production of squalene with promising antioxidant properties in callus cultures of *Nilgirianthus ciliatus*. Ind. Crops Prod..

[64] Kousalya L., Bai V.N. (2016). Effect of growth regulators on rapid micropropagation and antioxidant activity of *Canscora decussata* (Roxb.) Roem. & Schult.–A threatened medicinal plant. Asian Pac. J. Reprod..

[65] Gul M.Z., Ahmad F., Kondapi A.K., Qureshi I.A., Ghazi I.A. (2013). Antioxidant and antiproliferative activities of *Abrus precatorius* leaf extracts-an in vitro study. BMC Complement. Altern. Med..

[66] Upadhyay R., Chaurasia J.K., Tiwari K.N., Singh K. (2013). Comparative antioxidant study of stem and stem induced callus of *Phyllanthus fraternus* Webster—An important antiviral and hepatoprotective plant. Appl. Biochem. Biotechnol..

[67] Siswadi S., Saragih G.S. (2021). Phytochemical analysis of bioactive compounds in ethanolic extract of *Sterculia quadrifida* R. Br. AIP Conf. Proc..

[68] Hoagland D.R., Arnon D.I. (1950). The Water-Culture Method for Growing Plants without Soil.

[69] Murashige T., Skoog F. (1962). A revised medium for rapid growth and bio assays with tobacco tissue cultures. Physiol. Plant..

[70] Cheng T., Xu C., Lei L., Li C., Zhang Y., Zhou S. (2016). Barcoding the kingdom Plantae: New PCR primers for ITS regions of plants with improved universality and specificity. Mol. Ecol. Resour..

[71] Dpooležel J., Binarová P., Lcretti S. (1989). Analysis of nuclear DNA content in plant cells by flow cytometry. Biol. Plant..

[72] Bennett M.D., Smith J. (1976). Nuclear DNA amounts in angiosperms. Philos. Trans. R. Soc. Lond. B Biol. Sci..

[73] Dewanto V., Wu X., Adom K.K., Liu R.H. (2002). Thermal processing enhances the nutritional value of tomatoes by increasing total antioxidant activity. J. Agric. Food Chem..

[74] Ordonez A., Gomez J., Vattuone M. (2006). Antioxidant activities of *Sechium edule* (Jacq.) Swartz extracts. Food Chem..

