# Supplementary material for: Genome Estimation and Phytochemical Compound Identification in the Leaves and Callus of Abrus precatorius: A Locally Endangered Plant from the Flora of Saudi Arabia
Source: Plants (Basel). 2022 Feb 21;11(4):567. doi: 10.3390/plants11040567 (PMC8877254; doi:10.3390/plants11040567)
Supplement: Supplementary file 1 [file plants-11-00567-s001.zip › plants-1591339-supplementary.pdf]

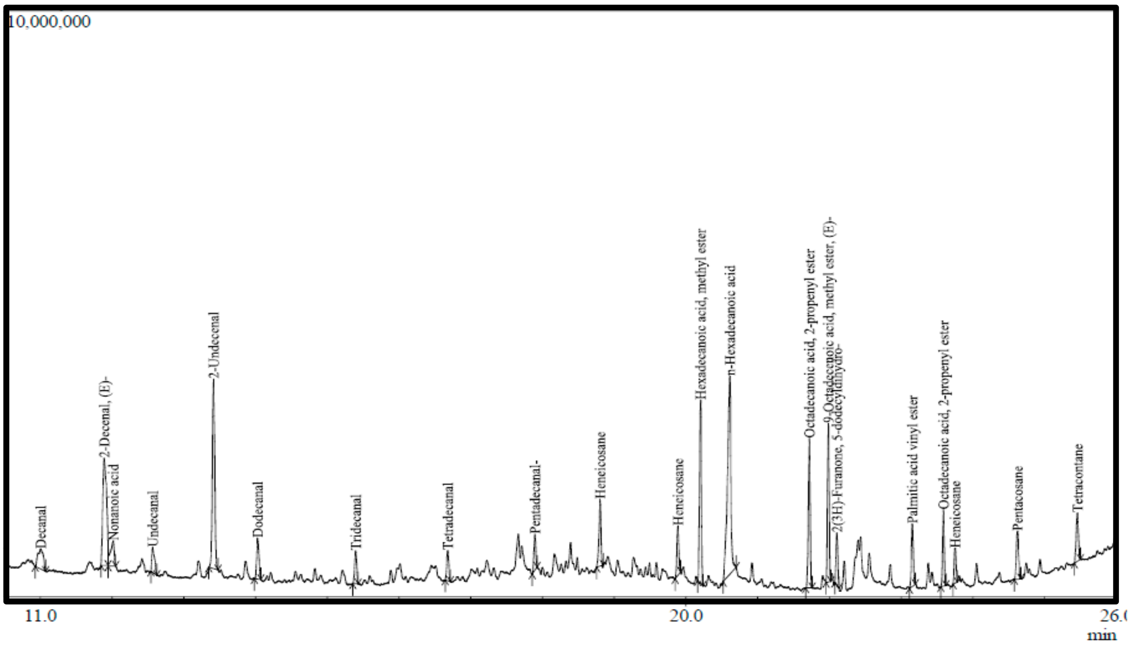

Figure S1. GC-MS chromatogram of the bioactive compounds present in the callus extract.

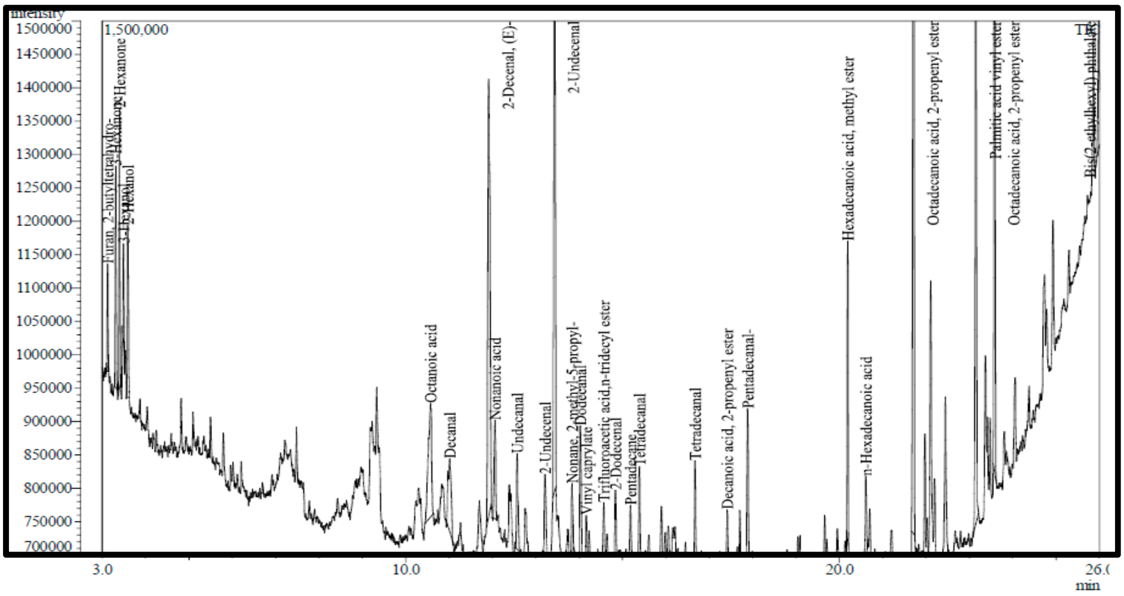

Figure S2. GC-MS chromatogram of the bioactive compounds present the leaves extract.
